# Sex Difference in Clinical Outcome of Patients With Implantable Cardioverter-defibrillator

**DOI:** 10.5812/cardiovascmed.5027

**Published:** 2013-02-24

**Authors:** Neshat Nazari, Sima Sayah, Nasrin Safavi, Mostafa Hekmat, Zahra Emkanjoo

**Affiliations:** 1 Cardiac Electrophysiology Research Center, Rajaie Cardiovascular Medical and Research Center, Tehran University of Medical Sciences, Tehran, IR Iran

**Keywords:** Sex Characteristics, Defibrillators, Implantable

## Abstract

**Background:**

Indications for the use of the Implantable Cardioverter-Defibrillator (ICD) have been greatly expanded in recent years, but the influence of sex on the efficacy of the ICD in eligible patients has still been remained unknown.

**Objectives:**

The aim of this study was to determine the impact of sex on the effectiveness of the ICD intervention for mortality and appropriate events.

**Materials and Methods:**

This retrospective study was conducted on the outcome of the ICD therapy in 443 patients, including 341 men with a mean age of 55 ± 16 years and 102 women with a mean age of 54 ± 17 years, in our center between April 2001 and February 2007. Sex-specific cumulative probabilities of outcomes concerning mortality and appropriate ICD intervention were evaluated for the patients.

**Results:**

Among the 443 patients, enrolled in this study, the women and men had a mean left ventricular ejection fraction of 35 ± 14.8% and 30 ± 13.5%, respectively (P = 0.03). Ischemic heart disease was more frequent in the men than the women (P = 0.0001).The average follow-up period was 3 years. Test for an interaction between sex and the ICD treatment regarding total mortality was not significant (23 men and 6 women). Additionally, there was no significant difference in appropriate events between the women and men (129 men and 33 women).

**Conclusions:**

While women were significantly less likely than the men to receive the ICD therapy, no conclusive evidence could be found for the impact of sex factor on the effectiveness of the ICD intervention with respect to mortality and appropriate events.

## 1. Background

Cardiovascular diseases continue to remain a major cause of death worldwide. For all sustained improvements in the prevention and treatment of cardiovascular diseases in recent decades, cardiovascular morbidity and mortality rates are still unacceptably high. Health disparities can be defined as health status differences on account of sex (1). Medicare coverage of the Implantable Cardioverter-Defibrillator (ICD) has enjoyed considerable expansion recently; nevertheless, most of eligible beneficiaries have not still received the ICD intervention. Previous studies have documented important sex differences regarding ICD use (1-3). It is plausible that because women have a documented lower risk of sudden cardiac death (4) and women with congestive heart failure have lower mortality (5-7) and sudden cardiac death rates, (8) they may not benefit to the same degree as men. Moreover, the available data on the benefits of the ICD therapy in women is still a matter of controversy (9,10).

## 2. Objectives

In the present study, we assessed the possible impact of gender differences on the efficacy of the ICD intervention regarding mortality and appropriate events.

## 3. Materials and Methods

This retrospective cohort study performed observational analysis of 443 patients who underwent initial ICD implantation for any cause between April 2001 and February 2007. All the patients received third-generation ICDs with biphasic shock waveforms and non-thoracotomy implants. Device programming was individualized according to the patients’ underlying cardiac disease and tachycardia characteristics. Clinical and demographic data, including age, gender, left ventricular ejection fraction (LVEF), New York Heart Association (NYHA) classification, underlying heart diseases, presence of coronary artery disease (CAD) as well as its risk factors [i.e. hypertension (HTN), smoking, diabetes, and dyslipidemia], and medications, were collected. Enrollment was commenced in 2001, and the patients were followed up at 3- to 6-month intervals for an average of 3 years. All the stored electrograms were classified as appropriate events if they documented a ventricular tachyarrhythmia and inappropriate events if the therapy was for sinus tachycardia, atrial fibrillation, or other supraventricular tachycardias. The primary end points of the study were mortality and appropriate events and their differences for sex factor.

Means or proportions for the baseline demographic and clinical variables were calculated for the men and women. The chi-square or fisher’s exact test and student’s t-test were used to compare the categorical and continuous variables. P ≤ 0.05 were considered statistically significant. The statistical analyses were conducted using SPSS 17 for Windows (SPSS Inc., Chicago, Illinois).

## 4. Results

The characteristics of the 443 patients, including 341 (77%) men with a mean age of 55 ± 16 years and 102 (23%) women with a mean age of 54 ± 17 years, are summarized inTable 1. The mean LVEF was 30 ± 13.5% for the men and 35 ± 14.8% for the women. The male patients had a significantly lower LVEF (P = 0.03) than their female counterparts, but their NYHA classification showed no more advanced stages of heart failure compared to women. Severe LV dysfunction (defined as LVEF < 25%) was seen in 130 (38%) men and 32 (31%) women, and the etiology of cardiomyopathy tended significantly to be ischemic in the men (P = 0.0001) and non-ischemic in the women. 72% of the men and 77% of the women had an ICD for secondary prevention of sudden cardiac death (P = 0.25). The patients who received the ICD implantation for secondary prevention had a higher risk of receiving an appropriate ICD therapy than those who received the ICD implantation for primary prevention. 25% of the patients who received implantation for primary prevention received appropriate ICD therapies compared to 40.7% of the patients who received implantation for secondary prevention (P = 0.004).

**Table 1.** Clinical and Demographic Characteristics by Gender

Parameter	Males, n=341	Females, n=102	P Value
**Age, Years**	55 ± 16	54 ± 17	NS
**LVEF ** ^**a**^ **, %**	30 ± 13.5	35 ± 14.8	0.03
**Ischemic Etiology**	238 (69.8 %)	34 (33.3 %)	0.0001
**NYHA ** ^**a**^ ** Class III, IV**	32 (9.4 %)	13 (12.7 %)	NS
**DM ** ^**a**^	51 (15 %)	76 (74.5 %)	NS
**Applied Therapies**	129 (37.8 %)	33 (32.4 %)	NS
**VF ** ^**a**^	28 (8.2 %)	20 (19.6 %)	0.002
**Mortality**	23 (6.7 %)	6 (5.9 %)	NS
**Smoker**	129 (37.8 %)	4 (3.9 %)	0.0001
**HTN ** ^**a**^	71 (20.8 %)	32 (31.4 %)	0.032
**History of MI ** ^**a**^	141 (41.3 %)	26 (25.5 %)	0.004
**History of CABG ** ^**a**^	93 (27.3 %)	6 (5.9 %)	0.0001
**Primary Prevention**	96 (28.2 %)	22 (21.6 %)	NS
**Secondary Prevention**	245 (71.8%)	79 (77.5%)	NS
**Electrical storm**	38 (11.1 %)	12 (11.8 %)	NS
**Antiarrhythmic Drug**	108 (31.7 %)	30 (29.4 %)	NS

^a^ Abbreviations: CABG: Coronary Artery Bypass Surgery, DM: Diabetes Mellitus, HTN: Hypertension, LVEF: Left Ventricular Ejection Fraction, MI: Myocardial Infarction, NYHA: New York Heart Association, VF: Ventricular Fibrillation

At enrollment, the women were significantly more likely to have HTN (P = 0.03), whereas the men were significantly more likely to be smokers and have a history of myocardial infarction and coronary artery bypass graft surgery (P = 0.0001, 0.004, and 0.0001, respectively). For other coronary risk factors such as diabetes and dyslipidemia, there was neither a significant difference between the two groups, nor between the male and female patients regarding medical treatment except for Aspirin use, which was significantly more frequent in the men (P = 0.02). The mean follow-up duration was 36 months.

There was a trend for the women to have fewer appropriate ICD interventions for ventricular arrhythmia than men; the difference, however, was statistically insignificant. Of all ventricular arrhythmias, the women had ventricular fibrillation (VF) more often (P = 0.002) compared with men. Also, appropriate events occurred more significantly in our diabetic patients (P = 0.005) and less significantly in our beta-blocker users (P = 0.048). Overall, 23 (6.7%) of the men and 6 (5.8%) of the women died during the trial. Aspirin users had lower mortality rate (P = 0.03); however; the test findings for an association between gender and mortality was not significant.

## 5. Discussion

As reported previously, women in the SCD-HeFT trial appeared to benefit from the ICD therapy less than men (11). On the other hand, the hazard ratio for the ICD therapy in women is known to have very wide confidence limits. These suggest that it would be improper to conclude that the ICD therapy is less effective on a relative basis in women than in men (12). Previous studies have demonstrated that women have a lower rate of sudden cardiac death than men, (12) which may explain their less survival benefits from the ICD therapy (10). Reasons for the lower incidence of sudden cardiac death in women are unclear, but these lower rates may be due to hormonal influences or gender differences in susceptibility to arrhythmia triggers (12). Healthy women have longer QT intervals and are more prone to drug-induced proarrhythmia than men, yet those given an ICD for ischemic cardiomyopathy have fewer episodes of VT/VF than men (13). The existing literature on the subject contains a wide array of conflicting results (14,15). In the current study, there was no gender difference regarding the incidence of sudden cardiac death. Previous trials have questioned the benefits of the ICD therapy in women. However, all these trials included an insufficient number of female subjects, which raises the possibility of gender bias (16). There were no gender differences in the delivery of appropriate ICD therapy during the follow-up period in the present study. More examples of contradictory results in the studies performed include the association between the female gender and an increased risk of ICD shocks reported in two investigations (14,17) and a decreased risk of shocks in two other studies (15,18). Some investigators have reported no gender differences in appropriate ICD therapy during follow-up (12,19). The reasons for these differences are unclear; but they might be related to the small female sample size or merely a matter of pure chance. Considering recent trials that have suggested no gender differences in the outcome of patients (12), we found no significant differences with respect to total mortality between our two groups of women and men. Our male and female patients, who received ICDs, had similar rates of appropriate events and mortality. In our study population, gender did not appear to be an important risk factor for sudden cardiac death.
